# New Path to Recovery and Well-Being: Cross-Sectional Study on WeChat Use and Endorsement of WeChat-Based mHealth Among People Living With Schizophrenia in China

**DOI:** 10.2196/18663

**Published:** 2020-09-18

**Authors:** Yu Yu, Yilu Li, Tongxin Li, Shijun Xi, Xi Xiao, Shuiyuan Xiao, Jacob Kraemer Tebes

**Affiliations:** 1 Department of Social Medicine and Health Management Xiangya School of Public Health, Central South University Changsha China; 2 Division of Prevention and Community Research Department of Psychiatry, Yale School of Medicine New Haven, CT United States; 3 Department of Psychiatry Changsha Psychiatric Hospital Changsha China

**Keywords:** WeChat, mHealth, schizophrenia, China, symptoms, functioning, recovery, quality of life, well-being

## Abstract

**Background:**

The past few decades have seen an exponential increase in using mobile phones to support medical care (mobile health [mHealth]) among people living with psychosis worldwide, yet little is known about WeChat use and WeChat-based mHealth among people living with schizophrenia (PLS) in China.

**Objective:**

This study aims to assess WeChat use, endorsement of WeChat-based mHealth programs, and health related to WeChat use among PLS.

**Methods:**

We recruited a random sample of 400 PLS from 12 communities in Changsha City of Hunan Province, China. WeChat use was assessed using the adapted WeChat Use Intensity Questionnaire (WUIQ). We also compared psychiatric symptoms, functioning, disability, recovery, quality of life, and general well-being between WeChat users and nonusers using one-to-one propensity-score matching.

**Results:**

The WeChat use rate was 40.8% in this sample (163/400); 30.7% (50/163) had more than 50 WeChat friends and nearly half (81/163, 49.7%) spent more than half an hour on WeChat, a pattern similar to college students and the elderly. PLS also showed higher emotional connectedness to WeChat use than college students. About 80.4% (131/163) of PLS were willing to participate in a WeChat-based mHealth program, including psychoeducation (91/163, 55.8%), professional support (82/163, 50.3%), and peer support (67/163, 41.1%). Compared with nonusers, WeChat users were younger, better educated, and more likely to be employed. WeChat use was associated with improved health outcomes, including lower psychiatric symptoms, lower depression, higher functioning, better recovery, and higher quality of life.

**Conclusions:**

WeChat-based mHealth programs hold promise as an empowering tool to provide cost-effective interventions, to foster global recovery, and to improve both physical and mental well-being among PLS. WeChat and WeChat-based mHealth programs have the potential to offer a new path to recovery and well-being for PLS in China.

## Introduction

People living with schizophrenia (PLS) are individuals with a diagnosis of schizophrenia, which is a disturbance of thought, perception, and a blunting of affect, which can be characterized by 3 major symptoms: psychosis, cognitive dysfunction, and negative symptoms [1,2]. Recent decades have seen a growing prevalence of mobile phone technology and an increasing interest in using mobile phones to support medical care (mobile health [mHealth]) among PLS worldwide. Mounting evidence shows that PLS own mobile phones and are highly engaged with mHealth [3-5]. A recent meta-analysis shows that 81.4% of PLS owned a mobile phone at some time in the last 2 years, a rate similar to the 90% observed in the general population [3]. The use of mobile phones and the internet among PLS is also similar to that in the general population [5]. The wide accessibility of mobile phones makes mHealth a valuable intervention approach for PLS as it can provide cost-effective, nonstigmatizing, and ongoing support, while overcoming geographic and temporal constraints [3-5]. PLS generally express great interest in mHealth with most in favor of using mobile phones to enhance contact with services and support self-management [3].

A burgeoning literature has consistently demonstrated strong evidence for the feasibility, effectiveness, and efficacy of mHealth [4-9]. For instance, a recent literature review shows an overall retention rate of 92% (95% CI 82%-98%) in mHealth among PLS, as well as a range of potential benefits and satisfaction reported by users [4]. Numerous studies have also shown benefits of mHealth for PLS, such as increased access to health care, remote monitoring and tracking of functioning, the capacity for supplementing and augmenting traditional therapy, enhanced self-monitoring and self-management, increased self-esteem and empowerment, enhanced social interactions and social support, improved medication adherence, decreased stigma due to their condition, relapse prevention, and improved social functioning [6-8].

Given these benefits, mHealth presents new opportunities to promote recovery and well-being among PLS. Recovery is a multifaceted concept that involves the development of new meaning and purpose in one’s life as one grew beyond the catastrophic effects of mental illness [10]. For PLS, recovery is not just about symptom reduction and functional improvement, but more an appreciation and satisfaction with life, self-esteem, and improved social functioning [11]. A large number of mHealth programs have been found to promote recovery for people with mental illness, including PLS [12-17].

In China, the most prevalent mobile app is WeChat (literally: micro message) owned by Chinese tech giant Tencent [18]. First released in January 2011, WeChat quickly gathered momentum and popularity, and has become the most widely and extensively used mobile social networking app in China [19]. According to a recent 2019 quarterly report, WeChat boasts over 1.13 billion monthly active users across a wide range of age groups [20], with 93% of urban users logging into WeChat on a daily basis [19]. WeChat has similar characteristics to WhatsApp for message release in various formats (eg, texts, videos, voices, and images) [18]. Additionally, it is similar to Facebook’s Newsfeed by enabling members to post text messages, pictures, emojis, web pages, and even small videos to Moments and to give and get comments [18]. Furthermore, WeChat provides additional functions such as entertainment, shopping, payments, banking, and city services, such as paying traffic fines and booking transportation [18]. Collectively, WeChat has seamlessly infiltrated every aspect of daily life for people in China, including PLS, and has generated innovative ways for connection, communication, and interactions as an all-purpose multifunctional app. Because of its high popularity and multiple functions, WeChat has been increasingly utilized as a medium for health interventions, with acceptability, feasibility, and efficacy well-established in people with various health conditions [21-24]. However, to date, most studies have focused on WeChat users from the general population or people with physical illnesses. Although WeChat is being used by PLS, research examining WeChat use rate and endorsement of WeChat-based mHealth among PLS is lacking. Such information may inform the development of WeChat-based mHealth interventions for PLS in China.

The wide recognition of the extensive benefits of mHealth for PLS worldwide, and the lack of research on WeChat use and WeChat-based mHealth among PLS in China represent a significant knowledge gap relevant to mental health services research in China. As the growth in demand for mental health care exceeds the resources available to China’s mental health system, it is critical to develop more innovative and cost-effective methods of health care delivery. WeChat and WeChat-based mHealth hold great promise for improving mental health care delivery through extending the reach of services and supplementing existing models of care [3]. However, the potential benefits of WeChat-based mHealth among PLS and their families depend on information on levels of WeChat access and engagement for this population [3]. It is thus essential to learn more about whether and how PLS use WeChat and WeChat-based mHealth if relevant WeChat-based mHealth programs are developed.

This study was conducted to fill this research gap by examining WeChat use, endorsement of WeChat-based mHealth programs, and health outcomes of WeChat users in an urban community sample of PLS in China. Specifically, we first examined participants’ WeChat use rate and patterns and compared these with 2 college samples and 1 elderly sample. We then examined participants’ endorsement of WeChat-based mHealth programs by assessing their interest in joining various potential WeChat-based programs. Finally, we examined how WeChat use is related to clinical outcomes, personal recovery, and well-being for this population

## Methods

### Participants and Procedures

This was a cross-sectional study conducted in 12 community health centers from May 2019 to September 2019. All participants were recruited from China’s largest demonstration project in mental health services—the “686 Program”. The “686 Program” is aimed at integrating hospital and community services for serious mental illness, with a series of services provided including a monthly free medicine distribution to registered patients [25,26]. Our target population was adult people aged 18 or older with a diagnosis of schizophrenia by the Chinese Classification of Mental Disorders-3 (CCMD-3) or the International Classification of Diseases-10 (ICD-10), living with at least one family member, and able to read and communicate. Those who were younger than 18 years of age with diagnosis other than schizophrenia, living alone, or being unable to read or communicate effectively due to illiteracy or disability were excluded. Using prevalence of WeChat use as our primary outcome variable, sample size was calculated based on the following formula for cross-sectional study: N=Z^2^ × (P × [1–P])/E^2^. Assuming α=.05 (accordingly, Z=1.96), P (prevalence of WeChat use)=0.4, and E (error)=5%, we came with a sample size of 369. In this study, a total of 400 PLS completed the interviews, satisfying the sample size requirement. The sample had a mean age of 46.87 (SD 10.99; range 18-77) and was comparably distributed by gender. Most were unemployed (358/400, 89.5%) and with an education level of middle and high school (271/400, 67.8%). The largest proportion of participants were married or living with partners (172/400, 43.0%), followed by single status (150/400, 37.5%). Most PLS had a diagnosis of schizophrenia for over 10 years (347/400, 86.8%), with a mean duration of 21.42 (SD 10.62) years. Most PLS started treatment and took medication within 1 year of their diagnosis (357/400, 89.3%). All PLS received free standard and unified medication through the 686 Program, which mainly included risperidone, clozapine, and aripiprazole.

The study was approved by the Institutional Review Board of the Xiangya School of Public Health of Central South University. During the monthly free medicine distribution day, a research team of 3-5 psychiatrists went to each health center, where registered people with mental illness receive medication refills. A poster with detailed information about the study was posted in each health center to promote study participation. Individuals who expressed interest in participating in the study completed a clinical assessment about their current symptoms and functioning by 3 psychiatrists and a brief survey by the research team. All participants had the study explained to them and provided written informed consent before participating. Their responses were then checked by a quality control member of the team to ensure there were no inconsistencies or missing items. All participants were reimbursed with RMB 10 (US $1.4) in return for their time for participating.

### Measures

#### WeChat Use

WeChat use was assessed with the WeChat Use Intensity Questionnaire (WUIQ), as adapted by 2 studies (Wen et al [27] and Pang et al [28]) with minor modifications (as in Ellison et al’s [29]) from the original assessment tool on Facebook use intensity. It is a 7-item questionnaire measuring the infiltration of WeChat into everyday life and users’ emotional attachment to WeChat [27-29]. The first question asks about the number of total WeChat friends one has, with optional answers ranging from 1 (0-50 friends) to 5 (more than 200 friends). The second question asks about the amount of time a person spends on WeChat in a typical day, with options ranging from 1 (0-30 minutes) to 5 (more than 3 hours). The rest of the survey includes a 5-item scale of emotional attachment to WeChat that asks about attitudes toward WeChat use. Each item is rated on a 5-point Likert scale from 1 (totally disagree) to 5 (strongly agree). A WeChat intensity score is then calculated by averaging the total set of question scores. The WUIQ has been increasingly used in China due to the wide popularity of WeChat-based studies, with satisfactory psychometric properties reported [27-29]. In this study, the WUIQ showed good internal consistency, with a Cronbach α of .80.

#### Psychiatric Symptoms

Psychiatric symptoms were measured with the 18-item Brief Psychiatric Rating Scale (BPRS-18) to assess a set of common symptom characteristics in patients with psychiatric disorders [30]. It covers 5 domains of clinical symptoms: affect, positive symptoms, negative symptoms, resistance, and activation as proposed by Shafer [31]. Each item is rated on an 8-point Likert scale from 0 (not assessed), 1 (not at all) to 7 (extremely severe). The total score ranges from 0 to 126, with higher scores representing greater severity of symptoms. The BPRS-18 has been frequently used in schizophrenia research and has well-established psychometric properties [30,31]. In this study, the BPRS-18 showed good internal consistency, with a Cronbach α of .85.

#### Functioning

Participant functioning was assessed using the Global Assessment of Functioning (GAF) scale to measure a person’s psychological, social, and occupational functioning on a hypothetical continuum of mental health illness ranging from 1 to 100 [32], with examples provided for each 10-level interval. This 1-item scale, with higher scores indicating better functioning, has been widely used in clinical assessments and has satisfactory psychometric properties [33,34]. In this study we assessed the functional level of PLS over the past month.

#### Disability

The 12-item World Health Organization Disability Assessment Schedule 2.0 (WHODAS 2.0) [35] was used to measure participant’s disability and functional impairment. It covers 6 domains of functioning: cognition, mobility, self-care, getting along with people, life activities, and participation in society [35]. Each item is rated on a 5-point Likert scale from 0 (no difficulty) to 4 (extreme difficulty) to assess the level of difficulty experienced while performing the activities. The total score ranges from 0 to 48, with higher score representing higher level of disability [36]. The WHODAS 2.0 has been widely used in China with good psychometric performance established [37,38]. In this study, the WHODAS 2.0 showed good internal consistency, with a Cronbach α of .89.

#### Depression

Depression was assessed with the Patient Health Questionnaire-9 (PHQ-9) [39] to screen for depressive symptoms by asking whether respondents have experienced a series of depressive symptoms in the past 2 weeks. Each item is rated on a 4-point Likert scale from 0 (not at all) to 3 (nearly every day) to assess the severity degree of depression symptoms. The total score ranges from 0 to 27, with higher score indicating more depression. The Chinese version of PHQ-9 has also been widely shown to be both culturally acceptable and psychometrically valid [40,41]. In this study, the PHQ-9 showed good internal consistency, with a Cronbach α of .92.

#### Anxiety

Anxiety was assessed with the Generalized Anxiety Disorder Scale-7 (GAD-7) [42] by asking whether respondents have experienced a series of anxiety symptoms in the past 2 weeks. Each item is rated on a 4-point Likert scale from 0 (not at all) to 3 (nearly every day) to assess the severity degree of anxiety symptoms. The total score ranges from 0 to 27, with higher scores indicating more anxiety symptoms. The Chinese version of GAD-7 has also been widely shown to be both culturally acceptable and psychometrically valid [43,44]. In this study, the GAD-7 showed good internal consistency, with a Cronbach α of .96.

#### Recovery

Recovery was assessed with the Recovery Assessment Scale (RAS). The RAS is the most widely used scale for measuring a personal perspective on recovery globally, and originally included 5 factors. In this study, we used an 8-item short form of RAS (RAS-8) composed of 2 factors: (1) personal confidence and hope, and (2) no domination by symptoms [45]. Each item is rated on a 5-point Likert scale from 1 (strongly disagree) to 5 (strongly agree). The total score ranges from 8 to 40, with higher score indicating better perceived recovery. In this study, the RAS-8 showed good internal consistency, with a Cronbach α of .91.

#### Quality of Life and General Well-Being

Quality of life and general well-being were measured using the first 2 general questions from the World Health Organization Quality of Life Brief Scale (WHOQOL-BREF) [46]. WHOQOL-BREF is a generic cross-cultural instrument to measure quality of life and is available in more than 40 countries [47]. Quality of life was assessed by asking “How do you evaluate your quality of life in the past two weeks?” on a 5-point scale from 1 (very bad) to 5 (very good). General well-being was assessed by asking “Are you satisfied with your health status?” on a 5-point scale from 1 (very unsatisfied) to 5 (very satisfied).

### Statistical Analysis

Scales and indices were tested for reliability, and exploratory and summary statistics were computed for all variables. Data were examined for the presence of missing and influential values, as well as for outliers, skew, and kurtosis. Continuous variables were described using mean and standard deviation, and categorical variables were described using frequency and percentage. WeChat use patterns in this study were compared with those of other populations in other studies using the same or similar scale [27,48,49]. WeChat users and nonusers were compared for sociodemographics by a two-tailed unpaired *t* test for age and χ^2^ test for gender, marriage, education, and employment. We also compared a series of health outcomes (symptoms, functioning, disability, depression, anxiety, recovery, quality of life, and general well-being) between WeChat users and nonusers using propensity score matching. Propensity score matching is a widely used method for robust comparison of outcomes between groups by controlling for observed group differences [50]. To control for selection bias, the WeChat use group and nonuse group were 1:1 matched using an optimal matching algorithm on the propensity score. Optimal matching is chosen to retain the maximal number of matched pairs by minimizing the global propensity score distance with replacement, thus minimize sample size loss and improve study external validity [51]. For match tolerance, we used a default value of 1 × 10^–5^ to check the overlap assumption. Propensity scores were generated using logistic regression with the model variables of age, gender, marital status, education, and employment. Differences in health outcomes can be estimated by conditioning on the estimated propensity score, assuming no unmeasured confounders [50]. All data were analyzed using STATA version 16 (StataCorp).

## Results

### WeChat Use and Patterns

WeChat use rate was 40.8% in this sample (163/400). As shown in Table 1, among the 163 WeChat users, 50 (30.7%) had more than 50 WeChat friends and nearly half spent more than half an hour on WeChat. Among the 5 items of emotional attachment to WeChat subscale, the top-ranking item was “WeChat is part of my everyday activity” with a mean score of 3.61 (SD 0.94; range 0-5) and for which 58.9% (96/163) of participants indicated agreement; by contrast, the lowest-ranking item was “I feel out of touch when I haven’t logged onto WeChat for a day” with a mean score of 3.06 (SD 1.17; range 0-5) and for which 41.7% (68/163) of participants indicated agreement. These results indicate that WeChat has become an important part of daily life for a sizable portion of WeChat users with schizophrenia.

We also compared the WUIQ score of the current PLS sample with 3 other available samples—2 college student samples and 1 elderly sample [27,48,49] in China. PLS had fewer WeChat friends than college students (mean 1.52 vs 2.34/2.44), and the percentage of PLS with more than 50 WeChat friends was similar to that of the elderly (50/163, 30.7% vs 12/35, 34.3%). However, the time PLS spent on WeChat daily was comparable to college students (mean 2.08 vs 2.00/2.77), but the percentage of PLS spending more than 30 minutes daily on WeChat was lower than that of the elderly (81/163, 49.7% vs 27/35, 77.1%). Comparisons on emotional attachment to WeChat were only available for college students, but these showed that PLS generally reported higher emotional attachment to WeChat than college students, with higher mean scores in all 5 items of the emotional attachment to WeChat subscale. Overall, these results indicate that PLS had WeChat friend numbers similar to the elderly, spent time on WeChat similar to college students, and yet had higher emotional attachment to WeChat use than college students.

**Table 1:** Summary statistics for WeChat intensity score and comparison with other studies.

Variables		People living with schizophrenia (N=163) (this study)		Undergraduates and graduates (N=339) [27]		College students (N=508) [48]		Old persons aged ≥50 (N=35) [49]	
**Number of WeChat friends^a^**									
	Continuous, mean (SD)		1.52 (0.99)		2.34 (1.71)		2.44 (1.31)		—^b^
	≤50, n (%)		113 (69.33)		125 (36.87)		—		23 (65.71)
	>50, n (%)		50 (30.67)		214 (63.13)		—		12 (34.29)
**Time spent on WeChat daily^c^**									
	Continuous, mean (SD)		2.08 (1.38)		2.00 (1.47)		2.77 (1.51)		—
	≤30 minutes, n (%)		82 (50.31)		165 (48.67)		—		8 (22.86)
	>30 minutes, n (%)		81 (49.69)		174 (51.33)		—		27 (77.14)
WeChat is part of my everyday activity^d^, mean (SD)		3.61 (0.94)		3.61 (1.03)		3.48 (0.99)		—	
I am proud to tell people I am on WeChat^d^, mean (SD)		3.28 (1.18)		2.77 (1.16)		2.74 (0.86)		—	
I feel out of touch when I haven’t logged onto WeChat for a day^d^, mean (SD)		3.06 (1.17)		2.27 (1.13)		2.76 (1.11)		—	
I feel I am part of the WeChat community^d^, mean (SD)		3.31 (1.01)		3.10 (1.08)		3.04 (1.00)		—	
I would be sorry if WeChat shut down^d^, mean (SD)		3.28 (1.04)		2.93 (1.25)		3.09 (1.08)		—	
Total mean score for all 7 items, mean (SD)		2.83 (0.77)		2.72 (1.26)		2.98 (0.78)		—	

^a^Optional answers include: 1=50 or less; 2=51-100; 3=101-150; 4=151-200; 5=more than 200; in the table we reclassified the answers into 2 classes with 50 as cutoff.

^b^Not available.

^c^Optional answers include: 1=less than 30 minutes; 2=30-60 minutes; 3=1-2 hours; 4=2-3 hours; 5=more than 3 hours; in the table we reclassified the answers into 2 classes with 30 minutes as cutoff.

^d^Optional answers include: 1=strongly disagree; 2=disagree; 3=neutral; 4=agree; 5=strongly agree.

### Endorsement of WeChat-Based mHealth Programs

Table 2 presents participants’ endorsement of WeChat-based mHealth programs. Among all 163 participants, 131 (80.4%) indicated a willingness to participate in any kind of WeChat-based mHealth program. Among the 3 proposed WeChat-based mHealth programs, the most commonly endorsed was psychoeducation (91/163, 55.8%), followed by professional support (82/163, 50.3%) and peer support (67/163, 41.1%). As for the number of WeChat-based mHealth programs participants were willing to participate, the majority were endorsing 1 kind of program (60/163, 36.8%), followed by all 3 kinds (38/163, 23.3%), 2 kinds (33/163, 20.2%), and no program at all (32/163, 19.6%).

**Table 2:** Endorsement of WeChat-based mHealth programs (N=163).

Variables		n (%)	
**Are you willing to participate in WeChat-based mHealth programs?**			
	No	32 (19.6)	
	Yes	131 (80.4)	
**Are you willing to participate in WeChat-based psychoeducation?**			
	No	72 (44.2)	
	Yes	91 (55.8)	
**Are you willing to join WeChat-based peer support group?**			
	No	96 (58.9)	
	Yes	67 (41.1)	
**Are you willing to receive WeChat-based professional support?**			
	No	81 (49.7)	
	Yes	82 (50.3)	
**Number of WeChat-based mHealth programs that respondents were willing to participate**			
	0	32 (19.6)	
	1	60 (36.8)	
	2	33 (20.2)	
	3	38 (23.3)	

### Sociodemographics Comparison Between WeChat Users and Nonusers

Table 3 compares sociodemographics between WeChat users and nonusers by two-tailed unpaired *t* test for age and χ^2^ test for gender, marital status, education, and employment. Significant differences were found in age, education, and employment between WeChat users and nonusers. Compared with nonusers, WeChat users were younger (41.70 vs 50.42, *P*<.001), more educated (college and above: 41/163, 25.2% vs 13/237, 5.5%, *P*<.001), and more likely to be employed (27/163, 16.6% vs 15/237, 6.3%, *P*=.001).

**Table 3:** Sociodemographic comparison between WeChat users and nonusers.

Characteristic			All respondents (N=400)	WeChat users		*P* value^a^	
				No (N=237)	Yes (N=163)		
Age, mean (SD)			46.87 (10.99)	50.42 (0.67)	41.70 (0.77)	<.001^b^	
**Gender**						.611	
	Male, n (%)		200 (50.00)	121 (51.05)	79 (48.47)		
	Female, n (%)	200 (50.00)		116 (48.95)	84 (51.53)		
**Marriage**						.403	
	Single, n (%)	150 (37.50)		83 (35.02)	67 (41.10)		
	Married/cohabited, n (%)	172 (43.00)		104 (43.88)	68 (41.72)		
	Else^c^, n (%)	78 (19.50)		50 (21.10)	28 (17.18)		
**Education**						<.001^b^	
	Primary and below, n (%)	75 (18.75)		66 (27.85)	9 (5.52)		
	Middle and high, n (%)	271 (67.75)		158 (66.67)	113 (69.33)		
	College and above, n (%)	54 (13.50)		13 (5.49)	41 (25.15)		
**Employment**						.001^b^	
	Unemployed, n (%)	358 (89.50)		222 (93.67)	136 (83.44)		
	Employed, n (%)		42 (10.50)	15 (6.33)	27 (16.56)		

^a^Descriptive statistics were compared with chi-square tests for categorical variables (gender, marriage, education, and employment) and unpaired *t* test for continuous variable (age).

^b^Significance at *P*<.05 or *P*<.01.

^c^Else include divorced, separated, and widowed.

### Health Outcome Comparison Between WeChat Users and Nonusers

Table 4 compares health outcomes between WeChat users and nonusers by propensity score matching modeled on age, gender, marriage, education, and employment. For health outcomes, significant differences were found in psychiatric symptoms, functioning, depression, recovery, and quality of life. Compared with nonusers, WeChat users had lower scores in psychiatric symptoms (30.47 vs 34.40, *P*=.030) and depression (8.58 vs 9.19, *P*=.024), as well as higher scores in functioning (66.13 vs 59.26, *P*<.001), recovery (23.92 vs 17.30, *P*<.001), and quality of life (3.28 vs 2.96, *P*=.002). In addition, WeChat users had lower disability and higher general well-being scores than nonusers, but the differences only reached a trend level effect after matching. No significant difference was found in anxiety scores between WeChat users and nonusers.

In order to know if health outcomes of WeChat users are further affected by the number of WeChat friends and the time PLS spent on WeChat, we conducted similar comparisons of health outcomes by number of friends (with 50 as cutoff) and time spent on WeChat (with 30 minutes as cutoff) using propensity score matching. Our results showed the greater than 50 WeChat friend group had significantly better functioning than the less than or equal to 50 WeChat friend group (difference=4.61, *P*=.024); they also reported higher a recovery (difference=3.20, *P*=.078), but with only a trend effect. No significant differences on other health outcomes were observed between these WeChat friend groups and the daily WeChat use groups (>30 and ≤30 minutes daily; Results are shown in Appendix 1).

**Table 4:** Health outcome comparison between the WeChat use group and nonuse group.^a^

Characteristic	All respondents (N=400), mean (SD)	WeChat use group (A), (N=163), mean (SD)	Non-use full group (B) (N=237), mean (SD)	(C) = A–B, *P* value (before matching)	Non-use matched group (D) (N=163), mean (SD)	(E) = A–D, *P* value (after matching)
BPRS-18^b^	32.90 (11.43)	30.47 (10.41)	34.53 (11.81)	<.001^c^	34.40 (10.72)	.030^c^
WHODAS 2.0^d^	26.02 (10.22)	23.49 (9.03)	27.74 (10.63)	<.001^c^	27.10 (9.31)	.080^e^
GAF^f^	61.83 (13.58)	66.13 (13.09)	58.97 (13.18)	<.001^c^	59.26 (11.61)	<.001^c^
PHQ-9^g^	9.01 (7.54)	8.58 (7.48)	9.30 (7.58)	.352	9.19 (6.52)	.024^c^
GAD-7^h^	6.67 (6.43)	6.08 (6.17)	7.08 (6.58)	.133	6.20 (5.67)	.101
RAS-8^i^	20.29 (9.31)	23.92 (9.07)	17.80 (8.65)	<.001^c^	17.30 (8.17)	<.001^c^
QOL-1^j^	3.05 (0.90)	3.28 (0.89)	2.89 (0.88)	<.001^c^	2.96 (0.82)	.002^c^
QOL-2^j,e^	3.02 (0.95)	3.16 (0.96)	2.92 (0.94)	.014^c^	3.00 (0.87)	.065^e^

^a^Continuous variables were compared using independent 2-sample unpaired *t* test for unmatched samples and paired *t* test for matched sample.

^b^BPRS-18: the 18-item Brief Psychiatric Rating Scale

^c^Significant at *P*<.05 or *P*<.01.

^d^WHODAS 2.0: the 12-item World Health Organization Disability Assessment Schedule 2.0

^e^Trend effect at *P*<.10.

^f^GAF: Global Assessment of Functioning

^g^PHQ-9: Patient Health Questionnaire-9

^h^GAD-7: Generalized Anxiety Disorder Scale-7

^i^RAS: Recovery Assessment Scale

^j^QOL: quality of life

## Discussion

### Summary

This study provides a first examination of WeChat use patterns, endorsement of WeChat-based mHealth programs, and health outcomes related to WeChat use among an urban community sample of PLS in China. We also compared use patterns and intensity of use with comparable data available for other groups, such as for college students or an elderly sample. Our findings show a promising WeChat use rate of 40.8% (163/400) among this sample, whose WeChat friend number is similar to that of an elderly sample. Although PLS spent comparable time on WeChat to the college student samples, they showed a higher level of emotional connectedness to WeChat use than college students. About 80.4% (131/163) of PLS were willing to participate in any kind of WeChat-based mHealth programs, with psychoeducation being the most commonly endorsed program (91/163, 55.8%). Compared with nonusers, WeChat users were younger, better educated, and more likely to be employed. WeChat use was also associated with improved health outcomes, including lower psychiatric symptoms, lower depression, higher functioning, better recovery, and higher quality of life. This suggests that WeChat users among PLS may represent a higher functioning group that are particularly amenable to mHealth programs using WeChat.

### WeChat Use and Patterns

In this study, WeChat use rate was 40.8% (163/400) among PLS, a rate within the range of 27%-71% for social media use reported among PLS in various studies across the world [52-57]. Thus, WeChat use among PLS in China is consistent with other popular social media use rates among PLS reported globally. When compared with 2 college student samples and 1 elderly sample, PLS had similar number of friends to the elderly, and spent similar time on WeChat to college students, but reported stronger emotional connectedness to WeChat use. The results extend the growing body of research showing that people with serious mental illness, including schizophrenia, are heavy consumers of internet and social media content [5,58,59]. PLS’s high emotional connectedness to WeChat may be related to negative stereotypes attached to mental illness that disrupt direct social contacts but makes the internet and social media a potentially safer alternative to meet social needs [60]. As a result, the WeChat use rate and emotional connectedness to WeChat use among PLS suggests that WeChat may be a widely accepted and appealing platform for health interventions in China.

### Endorsement of WeChat-Based mHealth Programs

The finding that 80.4% (131/163) of PLS were willing to participate in any kind of WeChat-based mHealth programs is consistent with past literature showing high endorsement of mHealth intervention among PLS and other persons with other psychoses [3]. This finding indicates a wide acceptability of WeChat-based mHealth programs for people with mental illness in China. Among the 3 proposed WeChat-based mHealth programs, the most commonly endorsed was psychoeducation (91/163, 55.8%), followed by professional support (82/163, 50.3%), and peer support (67/163, 41.1%). These results are consistent with the literature showing psychoeducation, peer support, and professional support as the top 3 promising and feasible interventions to effectively improve prognosis and well-being among PLS [61-63]. These findings have implications for future WeChat-based mHealth programs to first assess participants’ preferences and then provide targeted interventions based on their preferences.

### WeChat Use and Sociodemographic

Consistent with most of previous studies, our study finds that WeChat users are generally of younger age, have higher education, and being employed than nonusers. That younger people are more likely to use WeChat in China is constituent with worldwide data that younger people are more likely to get online and use social media, such as Instagram, Snapchat, and Facebook [64-66]. That people with more education were more likely to use WeChat is also consistent with other research showing positive associations between higher education and social media use [5,66]. For instance, the most recent research by the Pew Research Center showed an internet use rate of 79% among people with a college degree, which is much higher than the rate of 64% among people with a high-school education or less [64]. In addition, employed people were more likely to use WeChat which may be indicative of their higher economic status, thus making mobile phones and WeChat more accessible to them in general, as evidence shows that availability and access to mHealth are accounted for by economic rather than disease factors [3].

### WeChat Use and Health Outcomes

We found generally better health outcomes in WeChat users than nonusers, including lower psychiatric symptoms, lower depression, higher functioning, better recovery, and higher quality of life. While higher number of WeChat friends was associated with higher functioning and better recovery, daily time spent on WeChat use showed no significant relationship to any of the health outcomes in this study. It seems like WeChat use alone, regardless of WeChat use intensity, was associated with better health outcomes. This finding aligns with the growing literature showing that social media use is associated with improved clinical and psychosocial outcomes among PLS and other persons with other psychoses, with reports of fewer symptoms, better functioning, better recovery, and better quality of life [52,58,67]. One potential mechanism of better health outcomes among WeChat users may be through a larger social network and more social support [18,27]. This hypothesis is partially supported by our findings showing higher functioning and better recovery among the over 50 WeChat friends group. Further research is still needed to directly measure and compare social networks and social support between WeChat users and nonusers. It is also likely that the association between WeChat use and health outcomes may influence each other in a bidirectional way. For instance, the positive association between social functioning and WeChat use corresponds to an underdeveloped yet important conceptualization of social media participation as a dimension of social functioning, as raised by Bjornestad et al [58]. In this recent review, Bjornestad et al [58] found that only 1 out of the total 58 identified social functioning measures included social media as a social activity. They thus proposed that social media use be included into social functioning measurement in the future to avoid inadequate clinical assessment of social functioning.

### Limitations

This study has several limitations. First, our sample was drawn from 12 urban communities and may not be generalizable to other locales such as rural communities, where mobile phone and WeChat access may be lower. As a result, the findings in this study may not capture the whole picture of social media use among PLS in China. Future research may benefit from conducting a nationally representative sample of WeChat users who are PLS. Second, when comparing WeChat use intensity, we used 2 college student samples and 1 elderly sample as comparison groups, instead of the general population or similar PLS samples. This was due to the lack of similar WeChat use intensity data on these populations. We expect more studies on WeChat use among various populations, including PLS using the WUIS in the future to allow for more cross-comparisons. Third, the cross-sectional study design did not make it possible to establish causality between WeChat use and health outcomes. Future research should examine WeChat use longitudinally and examine the relationship between use and well-being. Fourth, WeChat, like any other social media, may carry the potential risk of violating personal privacy and confidentiality, which should be taken into consideration when designing WeChat-based mHealth research and interventions. All participants in research or interventions should be made aware of the potential privacy issues pertaining to their WeChat data and provide informed consent prior to participation.

### Conclusions and Implications

This initial study provides new data on the relationship between social media use using WeChat, mHealth program interest, and characteristics of WeChat users (PLS) in China. Our findings show a promising WeChat use rate and wide acceptability of WeChat-based mHealth programs among this population. This finding has implications for enhancing the current community-based treatment of PLS in China to augment existing treatment programs with WeChat-based mHealth interventions. Such interventions hold promise for reaching a larger population with schizophrenia, especially in regions of the world where traditional resources are scarce [3]. We also found that WeChat use was related to a series of positive health outcomes including decreased symptoms and depression, as well as improved functioning, recovery, and quality of life. This finding offers early validation of the interest and opportunities for leveraging popular social media platforms, such as WeChat, for supporting the health and well-being of PLS. WeChat-based mHealth programs can be an empowering tool to provide cost-effective interventions, to foster recovery and to improve both physical and mental well-being among PLS. WeChat and WeChat-based mHealth programs have the potential to lead to a new path to recovery and well-being for PLS in China. Finally, future research should build on the findings from this study to develop a WeChat-based integrative family intervention program that includes 3 key components surveyed in this study: psychoeducation, peer support, and professional support. Such an intervention could provide support and training for PLS and family members to facilitate recovery and improve the well-being of PLS directly as well as indirectly through improved quality of care provided by family caregivers [68].

## Abbreviations

BPRS-18: the 18-item Brief Psychiatric Rating Scale
CCMD-3: Chinese Classification of Mental Disorders-3
GAD-7: Generalized Anxiety Disorder Scale-7
GAF: Global Assessment of Functioning
ICD-10: International Classification of Diseases-10
PHQ-9: Patient Health Questionnaire-9
PLS: people living with schizophrenia
RAS: Recovery Assessment Scale
WHODAS 2.0: the 12-item World Health Organization Disability Assessment Schedule 2.0
WHOQOL-BREF: World Health Organization Quality of Life Brief Scale
WUIQ: WeChat Use Intensity Questionnaire
